# Oxidative Stress, HSP70/HSP90 and eNOS/iNOS Serum Levels in Professional Divers during Hyperbaric Exposition

**DOI:** 10.3390/antiox11051008

**Published:** 2022-05-20

**Authors:** Jakub Szyller, Mariusz Kozakiewicz, Piotr Siermontowski, Dorota Kaczerska

**Affiliations:** 1 Division of Clinical Chemistry and Laboratory Hematology, Department of Medical Laboratory Diagnostics, Faculty of Pharmacy, Wroclaw Medical University, Borowska 211A Str., 50-556 Wroclaw, Poland; 2 Division of Biochemistry and Biogerontology, Department of Geriatrics, Faculty of Health Sciences, L. Rydygier Collegium Medicum, Dębowa 3 Str., 85-626 Bydgoszcz, Poland; markoz@cm.umk.pl; 3 Department of Underwater Works Technology, Faculty of Mechanical and Electrical Engineering, Polish Naval Academy, Śmidowicza 69 Str., 81-127 Gdynia, Poland; p.siermontowski@amw.gdynia.pl; 4 Department of Physiotherapy and Health Sciences, Faculty of Dietetics, Gdańsk College of Health, Pelplińska 7 Str., 80-335 Gdańsk, Poland; dorotakaczerska@tlen.pl

**Keywords:** oxidative stress, simulated diving, diving physiology, heat shock protein, nitric oxide synthase

## Abstract

Heat shock proteins (HSPs) have protective effects against oxidative stress and decompression sickness. Nitric oxide may reduce bubble formation during decompression and its activity is regulated by HSPs. A simulated dive can cause the HSP response. The aim of this study was to describe the effect of simulated dives on the antioxidant system, HSPs, and nitric oxide synthase response and demonste the relationship between the concentration of HSPs and the intensification of oxidative stress. A total of 20 healthy professional divers took part in training, consisting of simulated dry dives in a hyperbaric chamber and split into experiment I (30 m exposure, 400 kPa) and experiment II (60 m exposure, 700 kPa) over 24 h. The activities of superoxide dismutase (SOD), catalase (CAT), and glutathione peroxidase (GPx) and the concentrations of malondialdehyde (MDA), heat shock protein 70 (HSP70), heat shock protein 90 (HSP90), endothelial (eNOS) and inducible (iNOS) nitric oxide synthase were measured. Increases in the activity of SOD and MDA concentration were demonstrated. The activity of GPx depended on the dive profile. The HSP70 serum level in both experiments was significantly lower after the dives. The mean HSP90 level was significantly higher after the simulated dive at 60 m. A significant relationship between HSP concentration and SOD/GPx activity was demonstrated. eNOS concentration increased after 60 m exposure. No change in iNOS concentration was observed. In conclusions, the simulated dive significantly affected the antioxidant system, heat shock protein expression and nitric oxide synthase; however, the changes depend on the diving conditions. There is a relationship between the expression of HSPs and the intensity of oxidative stress.

## 1. Introduction

Diving is an underwater sport activity, but it can also be used for well defined, specific professional purposes, practiced by highly specialized divers. Simulated dives take place in a hyperbaric chamber, do not require physical effort from the diver and consist of changes in pressure in the chamber, corresponding to the pressure at a suitable depth underwater. This type of diving, among others, is used during diving training.

There are several classes of hazards inherent to the underwater environment (high ambient pressure, which could cause barotrauma [1], bubble formation risk and decompression sickness [2], hypothermia [3], etc.). There are also several physiological problems such as protection from hydrostatic pressure changes, changes in breathing gas density, the narcotic effect of inert gases and decompression sickness (DCS) [4]. The major effects of pressure changes depend on water depth. This is extremely important because the lungs may therefore experience significant changes in volume, especially with rapid changes in depth [5]. This may cause very important problems, particularly during an uncontrolled ascent, such as barotrauma or decompression illness (DCI).

During the dive, an increase in the partial pressures of gases (e.g., O_2_, N_2_, etc.) is observed [5]. Diving is a special form of activity which induces oxidative and nitrosative stress [6,7]. Under stressful or pathological conditions (e.g., oxidative stress), various elements such as heat shock proteins (HSPs) play a cytoprotective role [8,9,10]. Heat shock proteins are the most highly conserved stress response proteins over evolutionary history [11,12]. HSPs are multifunctional and closely interact with the antioxidant system and the nitric oxide generation system [13,14,15]. A deficiency or excess of antioxidants modulates the activation of heat shock transcription factor (HSF-1) and subsequent HSP70 biosynthesis [16]. HSF-1 can be activated by oxidative stress and increases the synthesis of protective HSPs.

The induction of HSP70 expression alleviates air embolism-induced lung injury and reduces the severity of DCS after hyperbaric exposure [17], which can be very useful in preventing DCS. The induction of HSPs may be also the cause of decreased bubble formation in all kinds of DCI as a result of the beneficial effects HSPs exert on endothelial cells and the interaction with the metabolic pathways of NO [18]. HSP70 also protect cells against damage induced by ischemia and inflammation and confers injury tolerance. HSP70 may reduce bubble formation, but the exact molecular mechanism is unknown. Perhaps the protective effect of HSP on the endothelium could be used to treat the new COVID-19 disease [19,20].

HSP90 can affect the proteolysis of endothelial nitric oxide synthase (eNOS, the primary source of NO in endothelial cells). The association between HSP90 and eNOS maintains the enzyme in a coupled state in which eNOS produces nitric oxide but not super-oxide anions [21]. An increase in HSP90-eNOS association leads to an increase in NO generation. The reaction between NO and O_2_^−^ leads to the generation of peroxynitrite (ONOO^−^), which can cause endothelial cell dysfunction and lead to eNOS uncoupling [22].

Inducible nitric oxide synthase (iNOS) contributes critically to inflammation and host defense. iNOS expression is induced by endotoxins and cytokines. HSP90 is essential for iNOS gene transactivation and HSP70 is required for IKK (IκB kinase) activation and STAT1/IRF-1 (signal transducer and activator of transcription 1/interferon regulatory factor 1) promoter binding amid iNOS gene transactivation [23,24]. A significant impact of hyperbaric exposure on endothelial function, NO and peroxide production was confirmed in earlier research by Kozakiewicz [25].

There is no detailed data on HSPs and NOS protein expression in people who dive and are exposed to high pressure. The available results relate mainly to research on cell lines. We decided to study the effect of simulated diving on human HSP70 (HSPA1), HSP90 (HSP90α, inducible isoform), eNOS and iNOS serum concentrations of healthy, professional and experienced firefighters. We attempted to investigate selected parameters of oxidative stress such as superoxide dismutase (SOD1, E.C. 1.15.1.1), catalase (CAT, EC 1.11.1.6), glutathione peroxidase (GPx, EC 1.11.1.9) and malondialdehyde (MDA) to determine the effect of simulated diving on the antioxidant system, relative lipid damage and assess the relationship between these parameters and the HSP concentration—the possible antioxidant factor.

## 2. Materials and Methods

### 2.1. Bioethics Commission

The Bioethics Commission of the Collegium Medicum in Bydgoszcz of Nicolaus Copernicus University in Torun (KB/402/2004) has agreed to carry out this study. The research has been complied with all the relevant national regulations, institutional policies and in accordance with the tenets of the Helsinki Declaration.

### 2.2. Subjects

A total of 20 healthy professional divers (firefighters) took part in this study (in experiments I and II—30 msw (meters of sea water) and 60 msw exposures). All participants were men aged 25–38 years old. Detailed data are presented in Table 1. All divers were not subjected to high-pressure exposure for at least 72 h before our hyperbaric studies. They also did not take any medications or consume alcohol (but not 72 h (min.) before the test) and are not cigarette smokers. All participants followed similar diets without supplements with antioxidants. Due to their work as professional firefighters, the participants were fit and did not report any problems with physical performance.

### 2.3. Protocol

**Figure 1:**
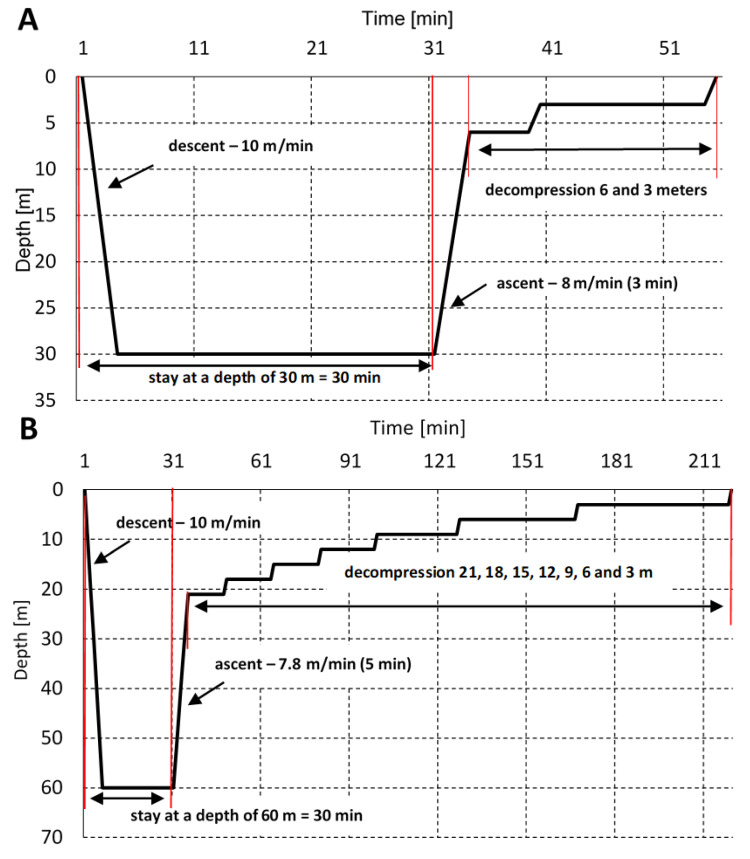
The dive profiles—30 m (**A**) and 60 m (**B**). The time of stay at depths of 30 and 60 m, including the time of the descent. Decompression stations are marked.

There were two studies undertaken where subjects were exposed to hyperbaric conditions in a hyperbaric chamber, simulating the first 30 m depth of sea water (max. 400 kPa (4 ATA, ~3000 mm Hg), which corresponds to ~630 mm Hg oxygen at max. depth; experiment I) and 60 m depth after 24 h (max. 700 kPa (7 ATA, ~5250 mm Hg), which corresponds to ~1102 mm Hg oxygen at max depth; experiment II), with oxygen decompression in both cases (pure oxygen was used for accelerated decompression). Decompression tables of the Polish Navy were used (Polish Navy 860/81). Exposure at 30 m lasted for a total of 54 min (with decompression and plateau time). Exposure at 60 m lasted for a total of 219 min. The profiles of exposures are presented in Figure 1. To reduce the risk of DCS symptoms (also popularly known as “bends”), 33 and 63 m decompression protocols were used. Hyperbaric exposure was carried out in cooperation with the Institute of Maritime and Tropical Medicine of the Military Institute of the Health Services in Gdynia, Poland. Exposure was conducted by a qualified medical and technical employee at the Institute of Diving Equipment and Underwater Technology of the Polish Naval Academy in Gdynia.

### 2.4. Blood Sampling

Initial blood samples were collected before exposure in the hyperbaric chamber. Immediately after blood collection, the divers performed a simulated exposition at a maximum depth of 30 or 60 m. The second blood collection was carried out immediately after decompression. All blood samples were drawn from the antecubital vein of subjects directly into vacuum tubes with silica as a clotting activator to obtain serum or with EDTA to obtain erythrocytes. Phlebotomy was performed only by qualified medical staff. After coagulation at a room temperature for 30 min, the fibrin cloth and the blood cells were removed by centrifugation for 10 min at 2000× *g*. The serum samples were frozen at −80 °C until assayed. Red blood cells were obtained from EDTA whole blood for the determination of enzyme activity.

### 2.5. Measurement and Analysis

The HSP70, HSP90, eNOS and iNOS serum levels were determined in duplicate by commercially available ELISA (enzyme-linked immunosorbent assay) kit (Cloud-Clone Corp., Houston, TX, USA), according to the manufacturer’s instructions. The microplates were washed with an automatic microplate washer (BioSan Microplate INTELIWASHER 3D-IW8, Biosan SIA, Rīga, Latvia). The absorbance of ELISA test results were read by a standard microplate reader (SPECTROstar Nano, BMG LabTech, Ortenberg, Germany) at 450 nm. The activities of SOD, CAT and GPx were determined in erythrocytes. The hemolysate was obtained by rinsing erythrocytes four times in 0.9% NaCl solution, and then the cells were lysed with cold deionized water. Due to the determination of enzyme activity in erythrocyte hemolysate, the results were converted into one gram of hemoglobin (to standardize the results). SOD was measured according to the method by Misra and Fridrovich (λ = 480 nm) [26], CAT according to Beers and Sizer’s method (λ = 240 nm) [27] and GPX using Paglia and Valentine’s (1967) method (λ = 340 nm) by Kozakiewicz in earlier research. MDA concentration was also determined in erythrocytes according to Placer et al.’s method (λ = 532 nm) [28].

### 2.6. Statistical Analysis

All statistical procedures were conducted using GraphPad Prism 8.0.1 Software (GraphPad Software, San Diego, CA, USA). All results are expressed as mean ± SD (standard deviation). A nonparametric Wilcoxon test was used to compare the pre-dive and post-dive HSP70, HSP90, and eNOS levels and pre-dive and post-dive SOD, CAT and GPx activities. A *p* < 0.05 was considered as significant. The results are presented as a percentage of the control value. Correlation analyses were also carried out.

## 3. Results

### 3.1. SOD Activity

**Figure 2:**
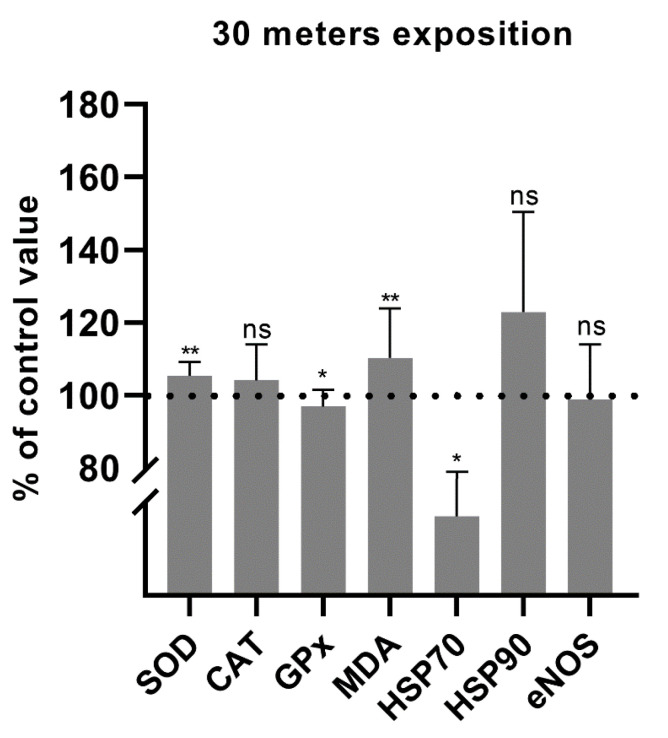
Percentage changes in SOD, CAT and GPx activities and in MDA, HSP70, HSP90, and eNOS concentrations after 30 m exposure (* *p* < 0.05, ** *p* < 0.01, ns = *p* > 0.05).

**Figure 3:**
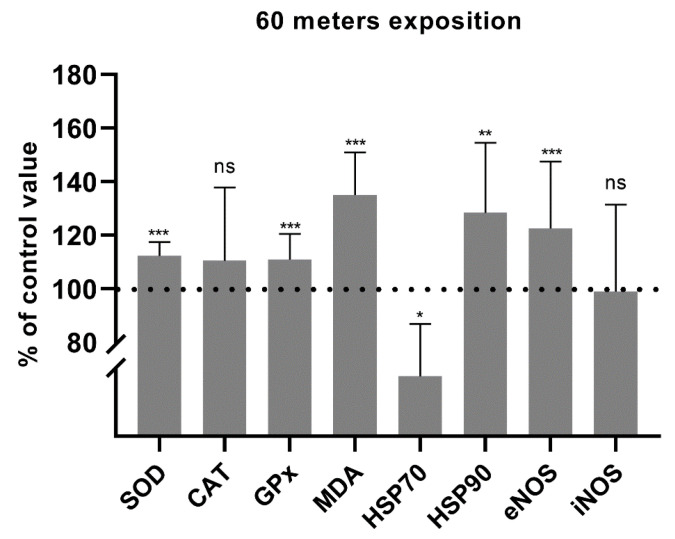
Percentage changes in SOD, CAT and GPx activities and in MDA, HSP70, HSP90, and eNOS concentrations after 60 m exposure (* *p* < 0.05, ** *p* < 0.01, *** *p* < 0.001, ns = *p* > 0.05).

**Figure 4:**
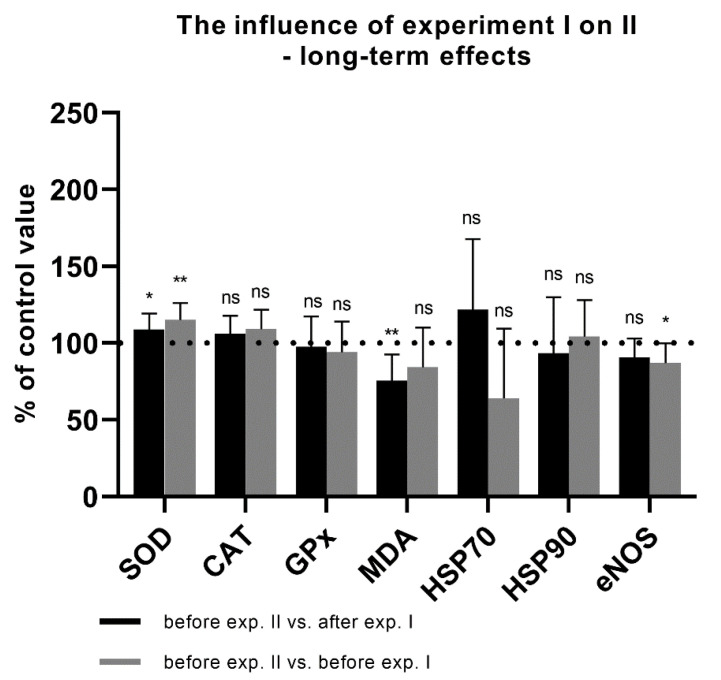
Long-term effects of experiment I. The activity or concentration of biochemical parameters before the exposure of 60 m in relation to the values before and after the first experiment I (exposure of 30 m) (* *p* < 0.05, ** *p* < 0.01, ns = *p* > 0.05).

The mean post-dive SOD activity in experiment I was 105.3% of the pre-dive value. This was statistically significant, with *p* = 0.001 (Figure 2). In experiment II, the mean post-dive SOD activity increased to 112.2%. This was statistically significant, with *p* < 0.0001 (Figure 3). There was also a significant increase in SOD activity before experiment II in relation to the values after experiment I from the previous day (108.0%; *p* = 0.026). An increase in SOD activity to 115.0% (*p* = 0.004) was also observed as compared to the values before experiment I (baseline value before hyperbaric exposures) (Figure 4). These data indicate an increase in antioxidant activity after hyperbaric exposures, related to oxidative stress.

### 3.2. CAT Activity

The mean post-dive CAT activity in experiment I was slightly higher (104.0%; *p* = 0.458) than the pre-dive value (Figure 2). In experiment II, the mean post-dive CAT activity was 110.5% (*p* = 0.779) of the control value (Figure 3). Due to the small study group, the results should be interpreted carefully. The influence of hyperbaria on CAT activity was not clearly determined in our study. A slight increase in CAT activity before experiment II in relation to I was observed but without statistical significance (*p* = 0.155). This value was also higher than the level before the first experiment, with borderline statistical significance (*p* = 0.050) (Figure 4).

### 3.3. GPx Activity

After experiment I, GPx activity decreased slightly to 96.8% (*p* = 0.040) (Figure 2). In experiment II, GPx activity increased to 111.0% (*p* < 0.0001) (Figure 3). These data indicate an increase in antioxidant activity after hyperbaric exposure only in experiment II, related to oxidative stress. There were no differences in the activity of GPx before experiment II in relation to the values obtained after experiment I or in relation to the baseline value before experiment I (Figure 4). These data indicate increased antioxidant activity following deeper (60 m) exposures.

### 3.4. MDA Concentration

Malondialdehyde concentration increased significantly to 110.2% after experiment I (*p* = 0.009) (Figure 2) and to 135.0% after experiment II (*p* < 0.0001) (Figure 3). This, together with the activity of antioxidant enzymes, indicates an increase in oxidative stress with lipid damage after both hyperbaric experiments. A significant decrease in MDA concentration (to 75.6%; *p* = 0.006) was demonstrated between the end of experiment I and the beginning of experiment II. A borderline significant decrease in MDA concentration was also observed as compared to the value before experiment I (84.4%; *p* = 0.050) (Figure 4).

### 3.5. HSP70 Serum Level

The mean post-dive HSP70 concentration in experiment I was 69.8% of the pre-dive value (*p* = 0.019) (Figure 2) and 71.9% in experiment II (*p* = 0.018) (Figure 3). Interestingly, in experiment II, a clear upward trend was observed between experiment I and before experiment II the next day (121.7%), but without statistical significance (*p* = 0.575). An insignificant decrease in the HSP70 concentration was also observed as compared to the value before experiment I (64.0%; *p* = 0.114) (Figure 4).

### 3.6. HSP70 and CAT Correlation

Despite the fact that no significant increase in CAT activity was demonstrated and there was a decrease in HSP70 concentration, an interesting correlation was observed between HSP70 and CAT. The greater the increase in CAT activity, the higher the HSP70 concentration (and the smaller decrease in HSP70 concentration after exposure at the same time). This suggests the role of HSP70 in oxidative stress.

### 3.7. HSP90 Serum Level

The serum HSP90 protein concentration after 30 m exposure did not change significantly, although a clear upward trend was observed (122.9%; *p* = 0.091) (Figure 2). HSP90 concentration after exposure to 60 m increased significantly to 122.5%, *p* = 0.003, smaller SD (Figure 3). This shows that hyperbaric exposure has an effect on the concentration of extracellular HSP90, especially longer, using higher pressures. There was no significant effect of long-term exposure of 30 m on the concentration of HSP90 (Figure 4).

### 3.8. HSP90 and SOD, GPx Correlation

**Figure 5:**
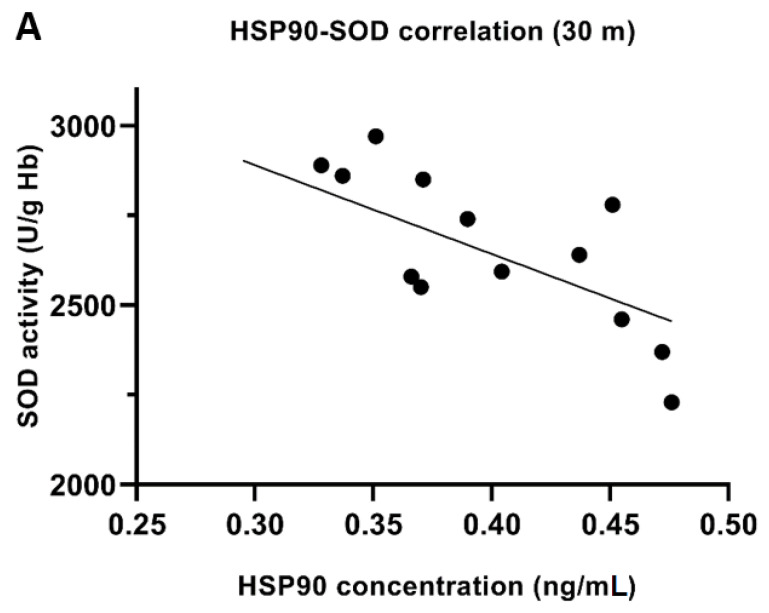

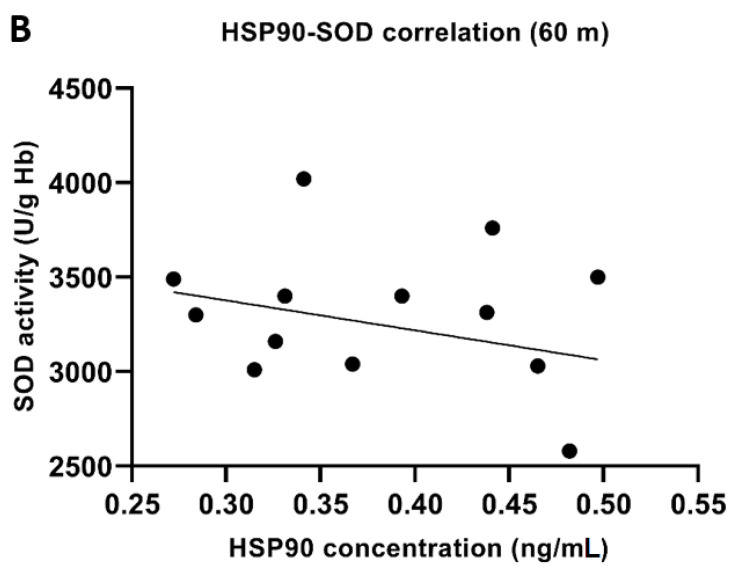
Correlation between HSP90 concentration (before diving) and SOD activity (after diving) in erythrocytes—30 m exposition (r = −0.58; *p* < 0.05) (**A**) and 60 m exposition (r = −0.27; *p* > 0.05) (**B**).

**Figure 6:**
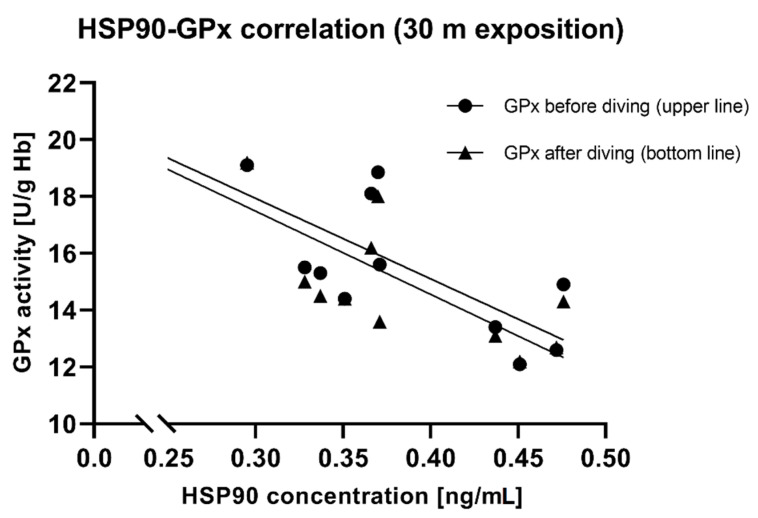
Correlation between HSP90 concentration and GPx activity in RBC (before diving, r = −0.79, *p* < 0.05; after diving r = −0.81, *p* < 0.05), 30 m exposition.

Pre-exposure HSP90 concentration correlated significantly negatively with SOD activity after 30 m exposure (r = −0.58, *p* < 0.05). The higher the concentration of HSP90 was before the dive, the lower SOD activity after the dive (Figure 5A). A similar relationship was observed after a 60 m dive, but without statistical significance (r = −0.27, *p* > 0.05) (Figure 5B). Pre-dive HSP90 also showed a negative correlation with GPx activity before and after diving (Figure 6). It suggests a protective role for HSP90 and/or a reduced release from cells and the reduction in extracellular HSP90 fraction.

### 3.9. eNOS Serum Level

Serum eNOS protein concentration after 30 m exposure did not change significantly (98.8%; *p* = 0.749) (Figure 2). In experiment II, the mean post-dive eNOS level was 122.5% of the pre-dive value (*p* < 0.001) (Figure 3). This suggests a significant effect of hyperbaric exposures on the serum concentration of eNOS and it may indirectly indicate an effect on endothelial function. There were no significant correlations between the concentration of heat shock proteins and eNOS. There was also no difference in the concentration of eNOS between the end of experiment I and the beginning of experiment II (90.6%; *p* = 0.050). A significant difference was found in relation to the initial value, before experiment I (87.0, *p* = 0.016) (Figure 4).

### 3.10. iNOS Serum Level

Serum iNOS protein concentration was determined only in experiment II. The concentration after 60 m exposure did not change significantly (99.0%; *p* = 0.927) (Figure 3). iNOS, under the tested conditions, probably does not play a significant role, unlike eNOS.

## 4. Discussion

Diving is a very specific model for studying human physiology. The impact of diving on oxidative stress has been confirmed in many studies [29,30]. Due to lack of data from real conditions, we decided to investigate the effect of simulated diving on HSPs and NOS expression among divers. This is the first time the tested oxidative stress parameters, HSPs and eNOS/iNOS concentration, have been described together in humans in real experimental simulated dives (apart from the study by Cialoni et al., where the authors assessed the concentrations of nitrates, nitrites and total plasma antioxidant capacity (TAC) before, during and after a single SCUBA dive). The available data indicated that HSPs may have an impact on the antioxidant system, the development of oxidative stress and the course of DCI [17,31,32].

Our results showed that the HSP70 serum level decreased after 30 m simulated diving, which is very interesting. A similar relationship was observed after 60 m diving. This is an interesting observation, as other authors of single publications noted the increase in HSP70 concentration in serum, in research on cell lines or, e.g., leukocytes [33,34,35]. Interestingly, an increase was observed before experiment II (in relation to I), suggesting a physiological return to baseline later. Both experiments reduced the concentration of HSP70, and the gap between them was perhaps too short for a complete return to the baseline. The initial decrease is perhaps related to reduced HSP expression or a decreased release from the cells as an extracellular fraction. The dive conditions may have had a large influence on the expression of HSP70. Djurhuus et al. show that the simulated dive at 26 bar, corresponding to a depth of 250 msw (meters of sea water) had a potentiating effect on HSP70 expression in human umbilical vein endothelial cells (HUVEC) [33]. However, the pressure was much higher and the experiment lasted for 24 h. The same authors show, in contrast to our results, that a simulated dive had no significant potentiating effect on the HSP90 level. We showed that the HSP90 level increased after diving, both at 30 and 60 m, while after 30 m it was not statistically significant. An increase in serum HSP concentration was also observed after simulated deep dives in Navy divers with Heliox gas mixture. Lee et al. observed that the HSP70 level increased after diving (not significantly; *p* = 0.07; *n* = 19), but only divers with less than 3 years of diving experiences showed a significant increase in HSP70 after simulated deep diving [34]. This may suggest that diving experience and acclimatization is important. It is very difficult to compare the results to other, identical studies. Each experiment was also designed differently and was carried out in completely different conditions. We can only draw general conclusions and mark the trend of changes in the expression of HSP. The decrease in the concentration of HSP70 is interesting. We could not determine the dynamics of changes after a certain time. The concentration increases later (HSP70 in experiment I vs. II) and the initial decrease may be a compensatory mechanism in response to high pressure and oxidative stress. In our study, blood was collected immediately after the end of the dives. The moment the material was collected for testing can therefore significantly affect the interpretation of the results. Taylor et al. observed a decrease in HSP70 expression in peripheral blood monocytes after simulated diving in a hyperbaric chamber in six healthy men (previously not exposed to hyperbaric exposures) [36]. Blood samples were taken 42 min after exposure and later after 4 h and 42 min. A decrease in HSP70 expression in monocytes was observed in both samples but in blood monocytes collected later, HSP70 expression was already close to the baseline observed before exposure [36]. This indicates a delayed increase in HSP70 concentration.

The expression of HSP90 has not been studied in terms of diving and oxidative stress. There is only publication available on this topic. We decided to study the changes in HSP90 concentration after dives and see if this protein can serve as an indicator of stress in divers. In one study from the USA, where US Navy sailors were tested, a 28% increase in HSP90 concentration was also observed on day 5 compared to day 1 after diving. Six-hour dives took place in a 4.57 m pool for 5 days [37]. For HSP90, we did not observe a significant long-term effect of the first exposure. Before the second experiment, the level of HSP90 was close to the baseline. This observation seems to be in line with the results obtained in US Navy sailors who only showed a slight increase in HSP90 after 5 days.

Our results showed that the hyperbaric exposures caused an increase in the activity of antioxidant enzymes such as SOD and GPx, although the activity of GPx decreased slightly after the first experiment. We showed no significant effect of the first experiment on GPx activity before the second (Figure 3). Except for this observation, which may be due to the shorter exposure time, this is in line with generally accepted knowledge. The reduction in GPx activity is consistent with the data obtained from people diving in real conditions to a depth of 40 m [38]. The increase in SOD activity before experiment II in relation to the values before and after experiment I from the previous day indicates the preconditioning effect of diving lasting for a long time (Figure 3). There was no significant increase in CAT activity, but this was most likely due to the small size of the study group or the conditions of our experiment; clear growth occurred later.

Despite this, an interesting correlation between HSP70 and CAT was observed. The greater the increase in CAT activity, the smaller the decrease in HSP70 concentration. This indicates the involvement of HSP70 in defense mechanisms against oxidative stress.

A correlation was found between HSP90 serum concentration before diving and SOD activity in erythrocytes after diving (Figure 5). The higher the pre-dive HSP90 concentration, the lower the post-dive SOD activity. We also showed a significant relationship between the HSP90 concentration and GPX activity. Pre-dive HSP90 showed a negative correlation with GPx activity before and after diving. People who had higher HSP90 levels had lower GPx activity. Our results show that HSPs and antioxidant enzymes were induced as a protective mechanism and HSPs may be involved in antioxidant protection. Xia et al. showed a negative correlation between HSP70 and SOD/GPx activity in gasoline workers exposed to oxidative stress [39]. The exact role of HSP in regulating oxidative stress is not fully understood, but for sure, HSP and antioxidant enzymes work together for cellular defense and ROS modulate HSP expression [40]. HSP70 can also regulate cellular redox status through modulating the activities of the GSH-related enzymes (e.g., GPx), which may be an important and critical mechanism for the cytoprotective effect [41]. HSP90 may have a similar effect. It should be noted that the exact role of HSP in serum (extracellular HSPs) is unknown. We used this material as it was easy to obtain and practically unstudied. As is known, gene expression need not be associated with protein biosynthesis. All data and results show that changes in HSP expression are useful markers for stress responses and may be useful for testing the barotolerance of divers.

When diving, divers are exposed to various external influences, which may affect cardiovascular function. We decided to investigate how hyperbaric exposures under the described conditions affect the concentrations of eNOS and iNOS in divers’ serum. There is practically no research available on this subject. Only after the simulating dive to 60 m did the eNOS level increase significantly; iNOS remained unchained. This increase in eNOS concentration seems to be in agreement with Kozakiewicz’s (the same conditions and the same group of firefighters) [25] and Theunissen et al.’s [38] observations, who observed an increase in circulating NO for the simulated or breath-hold diving (which causes significant hemodynamic changes), but not scuba diving at a depth of 20 m. Rahma et al. indicated that eNOS serum level decreased in the air diving group, whereas it increased in the Nitrox II (36% oxygen) group, at a higher oxygen concentration [42]. In our study, we did not find any changes in iNOS serum concentration. Sureda did not observe changes in neutrophil iNOS concentration after the dives at 50 m depth (for a total time of 35 min), but nitrite levels, which are indicative of iNOS activity, progressively increased after diving and recovery [7]. Importantly, despite the increase in eNOS protein concentration after 60 m exposure, the values before the second hyperbaric exposure were lower (Figure 4). Probably, during a simulated dive, the expression of eNOS increases; however, after its completion, the concentration of eNOS decreases even below the baseline values (maybe as a rebound effect).

In our experiment, we found no correlation between the antioxidant enzymes, MDA, HSP concentration and the NOS protein concentration. This is not a sign of their absence. HSP90 maintains eNOS enzyme in a coupled state, which is important for its biological activity and HSP70 is essential for the activation of the iNOS gene [21,23]. However, we did not show these relationships in blood serum after simulated dives under the described conditions. Cialoni et al. noticed an increase in serum NO concentration at the maximum depth (40 m), but did not observe it after surfacing, indicating very rapid changes in NO concentration [43]. Perhaps a similar relationship would be observed if blood samples were taken while divers were in a hyperbaric chamber.

It has been suggested that repeated compression and decompression cycles reduce diver susceptibility to DCS and dive training would reduce bubble formation and modulate endothelial function. Pontier indicated that repeated dives and regular physical activity reduce bubble formation and probably have a protective effect against DCS risk [44]. A NO-dependent change in the surface properties of the vascular endothelium favoring the elimination of gas micronuclei has been suggested to explain this protection against bubble formation. NO probably reduces the possibility of bubble precursors becoming attached to the vessel wall. Their formation is increased by NO inhibition in sedentary (but not in exercised) rats, suggesting the existence of other pathways, e.g., HSP-dependent ones [45]. It has been demonstrated that heat shock pre-treatments before diving enhanced the expression of HSP70 and can protect rats from lung injury due to air embolism [17]. The link between ROS, HSP, and NOS and endothelial function can also play a role.

Our study was based on a simulated dive model and it does not include the effect of immersion in water, as for SCUBA dives. This is probably the main reason for the differences between the results of our study and the results obtained by other authors, where divers were examined after real dives with water immersion. Our study, due to its very unique nature, was limited to a small study group; however, it is comparable to other studies. Thus, the obtained results must be interpreted carefully. We also did not investigate biochemical changes at the mRNA level. Therefore, we cannot conclude whether the changes result from a change in gene expression or, for example, from the protein release from cells.

## 5. Conclusions

The results of our research show that hyperbaric exposures significantly affect the expression of HSP and NOS, and indirectly the function of the endothelium. Diving in simulated conditions clearly increases the activity of antioxidant enzymes and has a significant impact on endothelial function. The changes in NO concentration, activity and NOS concentration, reported earlier, depend, as in the case of HSP, on the conditions of the experiments. It seems that increased HSP expression can be used as a stress biomarker in divers and plays a role in antioxidant mechanisms. Importantly, both HSP and NOS expression, the availability of free NO molecules and the interaction of HSP, NOS and antioxidant enzymes may play important roles in endothelial pathophysiology and the treatment of DCS or other diseases with endothelial dysfunction. Certainly, the changes depend on the diving conditions.

## Figures and Tables

**Table 1:** Demographic and medical characteristics of experimental group.

	N = 20		
	Mean ± SD	Median	Min–Max
**Age (years old)**	31.5 ± 4.1	32.0	25–38
**Height (cm)**	177.6 ± 6.6	176.5	165–190
**Body weight (kg)**	85.6 ± 12.8	81.4	68.9–103.7
**BMI (kg/m^2^)**	24.2 ± 2.3	23.9	22.5–28.4
**ALT (IU/L)**	28.1 ± 14.7	27.0	14.0–83.0
**Bilirubin (mg/dL)**	0.8 ± 0.3	0.7	0.2–1.3
**Glucose (mg/dL)**	96.1 ± 10.0	98.0	75.0–110.0
**Creatinine (mg/dL)**	0.9 ± 0.1	0.9	0.7–1.2
**eGFR (MDRD) (mL** **/min/1.73 m^2^)**	107.7 ± 17.0	108.4	74–143

## Data Availability

The data presented in this study are available on request from the corresponding author. The data are not publicly available due to the specific group of participants and their profession.

## References

[1] Russi E.W. (1998). Diving and the risk of barotrauma. Thorax.

[2] Cialoni D., Pieri M., Balestra C., Marroni A. (2017). Dive risk factors, gas bubble formation, and decompression illness in recreational SCUBA diving: Analysis of DAN Europe DSL data base. Front. Psychol..

[3] Smithuis J.W., Gips E., van Rees Vellinga T.P., Gaakeer M.I. (2016). Diving accidents: A cohort study from The Netherlands. Int. J. Emerg. Med..

[4] Bosco G., Rizzato A., Moon R.E., Camporesi E.M. (2018). Environmental physiology and diving medicine. Front. Psychol..

[5] Pendergast D.R., Moon R.E., Krasney J.J., Held H.E., Zamparo P. (2015). Human physiology in an aquatic environment. Compr. Physiol..

[6] Perovic A., Unic A., Dumic J. (2014). Recreational scuba diving: Negative or positive effects of oxidative and cardiovascular stress?. Biochem. Med..

[7] Sureda A., Batle J.M., Capó X., Martorell M., Córdova A., Tur J.A., Pons A. (2015). Scuba diving induces nitric oxide synthesis and the expression of inflammatory and regulatory genes of the immune response in neutrophils. Physiol. Genom..

[8] Fittipaldi S., Dimauro I., Mercatelli N., Caporossi D. (2014). Role of exercise-induced reactive oxygen species in the modulation of heat shock protein response. Free Radic. Res..

[9] Dimauro I., Mercatelli N., Caporossi D. (2016). Exercise-Induced ROS in heat shock proteins response. Free Radic. Biol. Med..

[10] Genest O., Wickner S., Doyle S.M. (2019). Hsp90 and Hsp70 Chaperones: Collaborators in protein remodeling. J. Biol. Chem..

[11] Lindquist S., Craig E.A. (1988). The Heat-Shock proteins. Annu. Rev. Genet..

[12] Radons J. (2016). The human HSP70 family of chaperones: Where do we stand?. Cell Stress Chaperones.

[13] Su C.Y., Chong K.Y., Owen O.E., Dillmann W.H., Chang C., Lai C.C. (1998). Constitutive and inducible Hsp70s are involved in oxidative resistance evoked by heat shock or ethanol. J. Mol. Cell. Cardiol..

[14] Dennog C., Radermacher P., Barnett Y.A., Speit G. (1999). Antioxidant status in humans after exposure to hyperbaric oxygen. Mutat. Res.-Fundam. Mol. Mech. Mutagen..

[15] Reeg S., Jung T., Castro J.P., Davies K.J.A., Henze A., Grune T. (2016). The molecular chaperone Hsp70 promotes the proteolytic removal of oxidatively damaged proteins by the proteasome. Free Radic. Biol. Med..

[16] Bianchi A., Moulin D., Hupont S., Koufany M., Netter P., Reboul P. (2014). Free radical biology and medicine oxidative stress-induced expression of HSP70 contributes to the inhibitory effect of 15d-PGJ 2 on Inducible prostaglandin pathway in chondrocytes. Free Radic. Biol. Med..

[17] Huang K.-L., Wu C.-P., Chen Y.-L., Kang B.-H., Lin Y.-C. (2015). Heat stress attenuates air bubble-induced acute lung injury: A novel mechanism of diving acclimatization. J. Appl. Physiol..

[18] Qing L., Yi H., Wang Y., Zhou Q., Ariyadewa D.K., Xu W. (2018). Benefits of hyperbaric oxygen pretreatment for decompression sickness in Bama pigs. J. Exp. Biol..

[19] Heck T.G., Ludwig M.S., Frizzo M.N., Rasia-Filho A.A., De Bittencourt P.I.H. (2020). Suppressed anti-inflammatory heat shock response in high-risk COVID-19 patients: Lessons from basic research (Inclusive Bats), light on conceivable therapies. Clin. Sci..

[20] Marino Gammazza A., Légaré S., Lo Bosco G., Fucarino A., Angileri F., Conway de Macario E., Macario A.J., Cappello F. (2020). Human molecular chaperones share with SARS-CoV-2 antigenic epitopes potentially capable of eliciting autoimmunity against endothelial cells: Possible role of molecular mimicry in COVID-19. Cell Stress Chaperones.

[21] Nitric-oxide E., Pritchard K.A., Ackerman A.W., Gross E.R., Stepp D.W., Shi Y., Fontana J.T., Baker J.E., Sessa W.C. (2001). Heat shock protein 90 mediates the balance of nitric oxide and superoxide anion from. J. Biol. Chem..

[22] Cassuto J., Dou H., Czikora I., Szabo A., Patel V.S., Kamath V., De Chantemele E.B., Feher A., Romero M.J., Bagi Z. (2014). Peroxynitrite disrupts endothelial caveolae leading to ENOS uncoupling and diminished flow-mediated dilation in coronary arterioles of diabetic patients. Diabetes.

[23] Luo S., Wang T., Qin H., Lei H., Xia Y. (2011). Obligatory Role of Heat Shock Protein 90 in INOS Induction. Am. J. Physiol. Physiol..

[24] Zhang L., Liu Q., Yuan X., Wang T., Luo S., Lei H., Xia Y. (2013). Requirement of heat shock protein 70 for inducible nitric oxide synthase induction. Cell. Signal..

[25] Kozakiewicz M., Kaczerska D., Ciesielska N. (2013). The effect of hyperbaric exposure on vascular endothelium’s capability of nitric oxide synthesis. Pol. Hyperb. Res..

[26] Misra H.P., Fridovich I. (1972). The Role of Superoxide Anion in the Autoxidation of Epinephrine and a Simple Assay for Superoxide Dismutase. J. Biol. Chem..

[27] BEERS R.F., SIZER I.W. (1952). A Spectrophotometric Method for Measuring the Breakdown of Hydrogen Peroxide by Catalase. J. Biol. Chem..

[28] Placer Z.A., Cushman L.L., Johnson B.C. (1966). Estimation of Product of Lipid Peroxidation (Malonyl Dialdehyde) in Biochemical Systems. Anal. Biochem..

[29] Kozakiewicz M., Kędziora-Kornatowska K., Pawluk H., Olszański R., Dąbrowiecki Z., Kornatowski T. (2011). Pro- and an-tioxidants processes in hyperbaric conditions. Pol. Hyperb. Res..

[30] Mrakic-sposta S., Vezzoli A., Rizzato A., Della Noce C., Malacrida S., Montorsi M., Paganini M., Cancellara P., Bosco G. (2019). Oxidative Stress Assessment in Breath-Hold Diving. Eur. J. Appl. Physiol..

[31] Medby C., Bye A., Wisløff U., Brubakk A.O. (2008). Heat Shock Increases Survival in Rats Exposed to Hyperbaric Pressure. Diving Hyperb. Med..

[32] Castagna O., Brisswalter J., Vallee N., Blatteau J.E. (2011). Endurance Exercise Immediately before Sea Diving Reduces Bubble Formation in Scuba Divers. Eur. J. Appl. Physiol..

[33] Djurhuus R., Nossum V., Lundsett N., Hovin W., Svardal A.M., Havnes M.B., Fismen L., Hjelde A., Brubakk A.O. (2010). Simulated Diving after Heat Stress Potentiates the Induction of Heat Shock Protein 70 and Elevates Glutathione in Human Endothelial Cells. Cell Stress Chaperones.

[34] Lee H.-C. (2009). Serum Heat Shock Protein after Simulated Deep Diving in Navy Divers. Chin. J. Physiol..

[35] Domoto H., Iwaya K., Ikomi F., Matsuo H., Tadano Y., Fujii S., Tachi K., Itoh Y., Sato M., Inoue K. (2016). Up-Regulation of Antioxidant Proteins in the Plasma Proteome during Saturation Diving: Unique Coincidence under Hypobaric Hypoxia. PLoS ONE.

[36] Taylor L., Midgley A.W., Sandstrom M.E. (2012). The Effect of the Hyperbaric Environment on Heat Shock Protein 72 Expression In Vivo. Res. Sports Med..

[37] Balogi Z., Multhoff G., Jensen T.K., Lloyd-Evans E., Yamashima T., Jäättelä M., Harwood J.L., Vígh L. (2019). Hsp70 Interactions with Membrane Lipids Regulate Cellular Functions in Health and Disease. Prog. Lipid Res..

[38] Theunissen S., Guerrero F., Sponsiello N., Cialoni D., Pieri M., Obeid G., Tillmans F., Papadopoulou V., Hemelryck W., Marroni A. (2013). Nitric Oxide-Related Endothelial Changes in Breath-Hold and Scuba Divers. Undersea Hyperb. Med..

[39] Xia B., Chen K., Lv Y., Huang D., Liu J., Liang G., Zhang L., Wang F., Su C., Zou Y. (2017). Increased Oxidative Stress and Plasma Hsp70 Levels among Gasoline Filling Station Attendants. Toxicol. Ind. Health.

[40] Gupta S.C., Siddique H.R., Mathur N., Vishwakarma A.L., Mishra R.K., Saxena D.K., Chowdhuri D.K. (2007). Induction of Hsp70, alterations in oxidative stress markers and apoptosis against dichlorvos exposure in transgenic drosophila melanogaster: Modulation by reactive oxygen species. Biochim. Biophys. Acta-Gen. Subj..

[41] Guo S., Wharton W., Moseley P., Shi H. (2007). Heat shock protein 70 regulates cellular redox status by modulating glutathione-related enzyme activities. Cell Stress Chaperones.

[42] Rahma I., Hati M., Suryokusumo G., Roestam A.W. (2019). Effects of Nitrox II during a Single Decompression Dive on Endothelial Nitric Oxide Synthase Expression and Flow-Mediated Dilation among Trained Male Divers. J. Nat. Sci. Biol. Med..

[43] Cialoni D., Brizzolari A., Samaja M., Pieri M., Marroni A. (2019). Altered Venous Blood Nitric Oxide Levels at Depth and Related Bubble Formation during Scuba Diving. Front. Physiol..

[44] Pontier J., Guerrero F., Castagna O. (2009). Bubble Formation and Endothelial Function Before and After 3 Months of Dive Training. Aviat. Space Environ. Med..

[45] Wisløff U., Richardson R.S., Brubakk A.O. (2003). NOS Inhibition Increases Bubble Formation and Reduces Survival in Sedentary but Not Exercised Rats. J. Physiol..

